# Imagine, Sing, Play- Combined Mental, Vocal and Physical Practice Improves Musical Performance

**DOI:** 10.3389/fpsyg.2021.757052

**Published:** 2021-10-25

**Authors:** Kristian Steenstrup, Niels Trusbak Haumann, Boris Kleber, Carles Camarasa, Peter Vuust, Bjørn Petersen

**Affiliations:** ^1^The Royal Academy of Music, Aarhus, Denmark; ^2^Center for Music in the Brain, Department of Clinical Medicine, Aarhus University, Aarhus, Denmark

**Keywords:** deliberate practice, auditory imagery, motor imagery, solfege, interleaved/random practice, varied practice, trumpet, brass pedagogy

## Abstract

Classical musicians face a high demand for flawless and expressive performance, leading to highly intensified practice activity. Whereas the advantage of using mental strategies is well documented in sports research, few studies have explored the efficacy of mental imagery and overt singing on musical instrumental learning. In this study, 50 classically trained trumpet students performed short unfamiliar pieces. Performances were recorded before and after applying four prescribed practice strategies which were (1) physical practice, (2) mental imagery, (3) overt singing with optional use of solfege, (4) a combination of 1, 2 and 3 or a control condition, no practice. Three experts independently assessed pitch and rhythm accuracy, sound quality, intonation, and musical expression in all recordings. We found higher gains in the overall performance, as well as in pitch accuracy for the physical practice, and the combined practice strategies, compared to no practice. Furthermore, only the combined strategy yielded a significant improvement in musical expression. Pitch performance improvement was positively correlated with previous solfege training and frequent use of random practice strategies. The findings highlight benefits from applying practice strategies that complement physical practice in music instrument practice in short term early stages of learning a new piece. The study may generalize to other forms of learning, involving cognitive processes and motor skills.

## Introduction

Within the classical music world there is a strong competition for few orchestral positions and resulting demand for high technical and flawless performance, and even this is not regarded sufficient as music performance at this level is considered meaningless without highly expressive and interpretative elements. For students at music academies this has led to a highly intensified practice activity to meet this demand. Similarly, professional musicians with a permanent position in a symphony orchestra experience an expectation of consistent high-level technical and musical performance often in a stressful psychological working environment (Kenny et al., 2014), spurring extended practicing. In both cases, such intensive and time-consuming activity challenges the physical apparatus and the mental wellbeing of the performer (Hagglund and Jacobs, 1996), potentially causing negative effects in the form of physical injuries, focal dystonia and overuse syndrome.

### Deliberate Practice

The notion that the amount of practicing, i.e., hours spent playing the instrument, determines a musician’s performance level is widespread. However, in a study that investigated the relative influence of practice quantity and quality, Ericsson et al. (1993) concluded that practice must be deliberate to fully support the development of expert music performance skills. According to this and later studies (Macnamara et al., 2014; Platz et al., 2014; Bonneville-Roussy and Bouffard, 2015), practice goals should be specific rather than being aimed at some vague general improvement.

Conventional physical practice, with prolonged repetition of certain movement patterns, may also carry the risk of a negative effect. In a study measuring the effect of different practice strategies on learning a difficult passage in piano literature, Duke et al. (2009) demonstrated that the strategies employed during practice determined more performance quality at retention, than frequency and duration of the pianists’ practice. Several studies have also pointed to the importance of differential practice, such as flexible, goal-oriented practice strategies (Shea and Morgan, 1979; Hall et al., 1994; Rohrer and Taylor, 2007; Rankin et al., 2009; Stambaugh, 2011).

Can physical practice be substituted or at least supplemented by alternative methods, such as mental practice (auditory and motor imagery) and overt singing, without compromising the overall performance quality? In the last few decades, studies on use-dependent neural plasticity using musical learning and performance as a model have generated important insights into human brain physiology, including higher cognitive processes implicated in controlling sensory, motor and emotional systems. In addition, strategies that aim to improve the effects of deliberate musical practice on instrumental and vocal performance have gained increasing scientific interest, providing novel insights into the cognitive basis of sensorimotor learning and corresponding benefits on learning efficacy, e.g., *mental practice* (Freymuth, 1993; Langheim et al., 2002; Highben and Palmer, 2004; Kleber et al., 2007; Bernardi et al., 2013), *varied practice* (Bangert et al., 2014), *interleaved practice* (Lin et al., 2013; Carter and Grahn, 2016), *practice with contralateral hand* (Grafton et al., 2002), and *use of solfege* (Wilson et al., 2006). Common for these practice methods, alternative to conventional physical practice, is the increased involvement of the dorsolateral prefrontal cortex (Grafton et al., 2002; Wilson et al., 2006; Kleber et al., 2007; Kantak et al., 2010; Lin et al., 2011), believed to be an essential component of the neural network responsible for procedural learning (Pascual-Leone et al., 1996) as it has extensive interconnections with regions involved in motor functions (Diamond, 2000; Lage et al., 2015).

### Auditory and Motor Imagery

Mental practice in music is most often simultaneously involving auditory and motor imagery. Auditory imagery is the use of mentally creating a vivid aural image of the music in question, the ability to clearly imagine the sound of the music being rehearsed. This pertains not only to pitch and rhythm, but infinite details of dynamics and timbre, articulation, vibrato, expression and style etc. (Herholz et al., 2008; Kuchenbuch et al., 2014; Regev et al., 2021).

A pioneering pedagogue in brass and wind methodology, Arnold Jacobs, taught from the principle that brass players must constantly “mentally sing” while playing to initiate precise vibration of their lips, thereby exciting the fundamental frequency and matching partials according to the harmonic series of the air column of the instrument (Frederiksen, 1996; Nelson, 2006). This becomes more crucial as the brass player moves into the high range where the same valve combination can trigger pitches that are just a major second apart, and in the extreme high range just one semitone. The educator Edwin Gordon (Gordon, 2011) defined a similar phenomenon and coined the term “audiation” to describe the ability to recall or create a mental image of the sound in response to memorized musical patterns. In a qualitative study, Trusheim (1991) interviewed 26 elite brass players in 5 leading US symphonic orchestras about their use of auditory imagery and found a highly developed faculty among the performers in imaging very subtle details of the rehearsed music as preparation for upcoming performances. The teaching of jazz improvisation, particularly the school of Lennie Tristano in the early history of jazz pedagogy, typically also includes auditory imagery (Jago, 2013). However, a large study by Miksza et al. (2018) did not find mental practice to be beneficial for skill acquisition in jazz improvisation.

More recent discoveries in neuroscience are providing insights beyond what was accessible during the lifetimes of Jacobs, Gordon and Tristano, i.e., into the probable neural functions that may confirm this ability of the human brain. Several studies, that explored mechanisms of neural plasticity in trained musicians, have established use-dependent adaptations within the sensory-motor system (Haueisen and Knösche, 2001; Lotze et al., 2003; Haslinger et al., 2005; Zatorre and Halpern, 2005; Bangert et al., 2006; Segado et al., 2018).

Of particular interest in the context of trumpet players, an fMRI-study by Gebel et al. (2013) involved trained pianists and trumpet players who engaged valves on an MRI-compatible model of a trumpet in relation to visually presented musical notes but without any auditory feedback. As opposed to pianists, trumpet players displayed an instrument training-specific co-activation increase in the left primary sensorimotor cortex in the somatotopic height of the lips, the trunk, the right cerebellar hemisphere and in the left primary auditory cortex, exhibiting the existence of an auditory-motor loop in motor tasks. This would implicate that auditory activity influences motor activity in trained musicians. Moreover, brain activity during auditory imagery involves cortical areas that overlap closely with those that are active during perception and motor planning, thus providing a possible explanation for why rehearsing music mentally may beneficially affect motor performance during playing (King, 2006).

Motor, or kinesthetic, imagery is considered a form of mental practice that apart from musicians also is employed by athletes (Ross, 1985; Suinn, 1997; Pascual-Leone et al., 1995; Ranganathan et al., 2004; Robin et al., 2007; Ridderinkhof and Brass, 2015). It is commonly described as the ability to imagine the correct movements of task-relevant body parts performing their desired function and for musicians this is very closely linked to simultaneously imagining the audible result of this activity. For a pianist, for instance, motor and auditory imagery would involve imagining the hands, fingers and the feet (for pedals) while mentally hearing the music these produce as a consequence of the desired movements. For a string player, motor imagery may involve the imagination of moving the fingers of the left hand on the fingerboard and the activity of the bow arm. As compared to pianists, string players, and percussionists, who mainly involve fine motor control of peripheral muscle groups, vocalists would have to involve body-core centered motor activity, involving muscles responsible for vital functions (e.g., breathing) and speech production (Kleber et al., 2007). Wind players in general and brass players in particular would have to engage both peripheral and body-core activity, such as fingers, the facial muscles around the mouth (the embouchure), the tongue, and the respiratory muscles.

In a study by Ross (1985), involving 30 college trombonists of North American universities, the participants played musical passages before and after practicing an assigned part using one of 5 different strategies: physical practice, mental imagery, a combination of physical and mental imagery, mental practice with simulated slide movement (dynamic motor imagery) and no practice (control). Recordings of the performances were assessed and scored according to gains in pitch, rhythm and articulation accuracy. The combination of physical and imagery practice strategies yielded significantly higher gains than any of the other strategies.

### Singing and Solfege

Another alternative method to physical practice is overtly singing the rehearsed music. This will tell the performer whether the aural image of the music is correct, not only in terms of pitch and rhythm, but also with regard to expressive musical parameters such as intensity, phrasing, articulation and even vibrato. Jacobs (Frederiksen, 1996; Nelson, 2006) emphasized this strategy as being crucial for brass players as this category of musicians control facial musculature into letting the lips vibrate the desired pitch analogously to vocalists who vibrate their vocal cords. Furthermore, he pointed to the influence of the lyricism and expressivity that may accompany actual singing.

**FIGURE 1:**
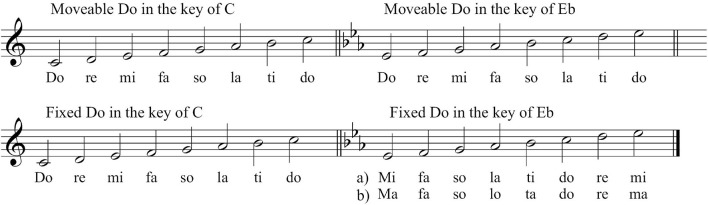
Top system: moveable Do in the key of C and the key of Eb. The syllables remain the same regardless of the key. Bottom system: Fixed Do in the same two keys. The tone names represent the sounding pitches. (a) Prevalent tradition in which syllables are unaltered regardless of flats or sharps. (b) Chromatic variant in which syllables are changed according to flattened or sharpened pitches.

A strong and widespread tradition in music education that aims at further supporting aural and sight-reading skills is solfege, which involves vocalizing the music using the Italian tone names: Do, Re, Mi, Fa, So, La, Ti, and Do. Currently there are two dominating ways of applying solfege, moveable Do and fixed Do. In the movable Do tradition, each solfege syllable corresponds to a scale degree. In fixed Do each syllable corresponds to the name of a note; consequently, Do is always a sounding C, Re always D, etc. This means that the name of each tone represents not a scale degree, but the actual sounding pitch (Figure 1) (McNaught, 1892).

Only a few studies have investigated the efficiency of the use of syllables to improve performance. Apostolaki (2012) found a positive effect of fixed-Do solfege on aural skills. The study, however, did not measure effects on the main instrument performance. Moreover, studies that explore the functionality of solfege are scarce, but one study pointed to linguistic-coding strategy for musical pitch retrieval used by fixed-Do trained musicians (Hsieh and Saberi, 2008). Other studies pointed to increased activity in the dorsolateral prefrontal cortex, suggesting increased involvement of working memory in pitch naming compared to singing the tones without applying syllables (Zatorre et al., 1994; Wilson et al., 2006). However, as actual singing is a much stronger component in the musical upbringing with solfege than in other forms of ear training, the familiarity with solfege may yield better singing ability and therefore provide a positive effect on the instrument performance (Davis, 1981; Rosenthal et al., 1988; Ohsawa, 2009).

### Random and Varied Practice

Random, or interleaved, practice is the manner of shuffling between shorter periods of types of skills practiced, (1-2-3-4, 1-2-3-4, 1-2-3-4, etc.) contrary to the more traditional use of blocked practice which involves longer sessions, practicing one type of skill (1-1-1-1, 2-2-2-2, 3-3-3-3 etc.) (Shea and Morgan, 1979; Rankin et al., 2009). Several studies have shown a robust positive effect of random as opposed to blocked practice across such diverse disciplines as baseball (Hall et al., 1994), mathematics (Rohrer and Taylor, 2007), and playing clarinet (Stambaugh, 2011; Carter and Grahn, 2016).

Also, varied, as opposed to constant, practice is characterized using a training regime that includes frequent changes of skill training so that the performer is constantly confronting new types of challenges by practicing a range of varying targets rather than by focusing on fixed repetitions of one target only (Kerr and Booth, 1978; Landin et al., 1993; Bangert et al., 2014). Types of varied practice in music practice may involve changes in rhythm, tempo, dynamics, timbre, keys, style, articulations or any other parameter to vary the immediate target.

The learning efficacy observed in random and varied practice is believed to be caused by high *contextual interference* because it requires greater cognitive effort during the execution of motor skills. Two hypotheses attempt to explain the contextual interference effect: (a) The elaborative-processing hypothesis developed by Shea and Zimny (1983) proposes that random practice requires a higher level of inter-task comparisons between trials than blocked practice, leading the learner to undergo further elaboration in memory; (b) the forgetting-reconstruction hypothesis suggests that random practice promotes the forgetfulness of a previously constructed action plan because the learner has to perform a different task during the next trial. It is the reconstruction of action plans upon return to prior tasks that leads to a stronger memory representation (Magill and Hall, 1990). However, these two hypotheses may not be mutually exclusive and may work in combination (Lage et al., 2015; Carter and Grahn, 2016).

### Focus of Attention

A number of studies across different athletic and musical disciplines (Zachry et al., 2005; Zentgraf et al., 2009; Duke et al., 2011; Wulf, 2013; Mornell and Wulf, 2019) have investigated the influence of focus of attention on training and performance outcomes. Typically, they distinguish between internal focus as being mentally occupied with parts of one’s own anatomy (for a trumpet player lips, tongue, breathing muscles), or external focus, thinking about the effect of the activity (musical elements, the sound of the music in the hall, where to direct the music or the wind etc.). The vast majority of studies (Wulf, 2013), whether in sports or musical performance, points to external focus as being most efficient and we hypothesized that there might be an influence when the participants are reporting familiarity with this aspect of practice and performance.

### Sleep and Meditation

Sleep has been implicated as a crucial factor in learning and consolidation of motor skills (Walker et al., 2002). Motor-sequence learning has shown that sleep after motor skill acquisition can trigger significant improvements in both performance speed and accuracy on a finger-tapping task, and that these overnight learning gains correlate with the amount of stage-2 non-rapid eye movement (NREM)- sleep. As we go through sleep stages during the night, stage 2-NREM-sleep happens particularly late in the night leading to the notion that motor skill learning will benefit from increased sleep duration. This effect will also show in daytime naps (Nishida and Walker, 2007). The influence of sleep has also been shown in consolidation of motor skills in music (Allen, 2007) and in auditory learning (Gaab et al., 2004). Meditation has also been seen to have a positive effect on motor learning (Immink, 2016) and we hypothesized that sleep and meditation habits could have an impact on practice outcome.

### Rationale and Hypotheses

The aim of the study was to investigate the effect of auditory and motor imagery and overt singing practice strategies on performance quality (Ross, 1985; Rosenthal et al., 1988; Coffman, 1990). The study involved 50 participants, all of whom played trumpet at academy level. In line with both previous research and pedagogical traditions, we hypothesized that a strategy in which physical practice is combined with motor and auditory imagery and overt singing will be equally effective in improving musical reproduction and quality as mere physical practice and more effective than strategies relying solely on either motor and auditory imagery or overt singing alone. Furthermore, we hypothesized that prior solfege, training and application of interleaved/varied practice regimes, focus of attention, sleep habits and meditation as self-reported in a participant survey, would impact the general performance level as well as the ability to improve during acquisition. The study adds to the Ross study from 1985 by including a substantially higher number of participants; testing the use of singing voice as a practice strategy; involving all participants in all strategies; including the participants’ self-reported data on musical training, practice habits and demographic information in relation to test data and involving an independent expert jury in assessing the pre- and post-test performances, assessing additional musical parameters such as intonation, sound quality and musical expression.

## Materials and Methods

### Participants

Fifty academy level trumpet students (mean age 23 year, *SD* = 2.9, range 17-28, 11 F, 39 M), studying at leading institutions in Germany, Denmark and Switzerland were recruited for the study. The participants were not required to do any preparation before the test and would have no knowledge of the nature of neither strategy nor study prior to the experiment. Following completion of the testing, the participants filled out a questionnaire containing 34 questions on autobiographical information such as age, handedness, musical background and training, use of practice strategies, lifestyle habits etc. (Table 1, see also Supplementary Material, Appendix 2 for complete list of questions). The experiment was approved by the internal review board (IRB) at The Danish Neuroscience Center at Aarhus University, Denmark. All volunteers gave written informed consent before participation in this study and data were stored in compliance with the guidelines of the Danish Data Protection Agency. Details regarding age, musical background and training, use of practice strategies and lifestyle habits are provided in Table 1.

**TABLE 1:** Participants’ demographic, musical background and lifestyle data (top) and musical practice habits data (bottom) as reported in the questionnaire.

Gender (f/m)	Mean age	Mean age at onset of music training	Mean age at onset of playing the trumpet	Mean age at onset of playing other instrument	Years of solfege or ear training (mean)	Total hrs. of playing age 4-17 (mean)	Learned solfege using solmization (yes/no)	Learned Fixed-do/Moveable-do/both	Possession of absolute pitch: yes/no/maybe	Hours of daily sleep including naps (mean)	Regular meditation yes/no
**Demographic, musical background and lifestyle data for participants (*N* = 50)**													
11/39	22.7 (2.8)	6.4 (1.9)	8.1 (2.1)	9.4 (5)	7.5 (5)	5844 (2325)	32/18	17/5/12	6/37/7	7.4 (0.9)	13/37

**Use of singing as part of practicing: every day/a few times per week/occasionally/never**	**Practice hrs. per day (mean)**	**Percentage of practice time = physical**				**Percentage of practice time = mental**				**Use of auditory imagery/motor imagery/both**	**Years using mental imagery practice (mean)**	**Use of blocked practice/random practice/both**	**Use of external focus/internal focus/both**
		**1-25**	**26-50**	**51-75**	**76-100**	**1-25**	**26-50**	**51-75**	**76-100**				

**Practice habits data for participants (*N* = 50)**													

29/14/6/1	3.1 (1)	4	12	25	9	23	20	7	0	15/31/4	2.5 (2.5)	13/21/16	26/3/21

*Values in brackets indicate standard deviation.*

### Materials

A pool of five unfamiliar etudes made up the musical material. The etudes were taken from “Develop Sight Reading” by Gaston Dufresne. All etudes had an approximate duration of 50 s and were of similar difficulty (see Supplementary Material, Appendix 4). All participants played B-flat trumpet in all the etudes. A prior pilot study had confirmed that the etudes represented a comparable sight-reading challenge across the high-level participants to avoid a ceiling effect. It was also taken into consideration that the tonal range should not be influencing muscle fatigue in the physical practice test.

### Procedures

**FIGURE 2:**
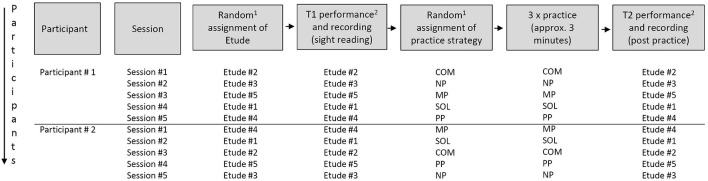
Experimental setup, exemplifying the potential course of five sessions for two participants. 50 participants took part in 5 sessions each, totaling to 250 sessions and 500 recordings. (1) Randomizations done with randomize.org. (2) With metronome.

A repeated measures design was applied to test the performance outcome with different practice strategies for each participant (see Figure 2). The participants’ tasks involved playing a total of five unfamiliar etudes before and after applying one of four different practice strategies, including physical practice, auditory/motor imagery, singing, and a combination of the above; or a fifth control condition with no practice. The trials were organized in five different sessions, dispersed over 1-3 days with breaks between trials, such that all participants played all 5 etudes and applied all 5 strategies in randomized order. The participants’ performances were recorded both before (T1) and after (T2) applying the respective practice strategy.

During T1-recordings, participants played the assigned etude while sight reading, accompanied by a metronome set at a predetermined tempo. After recording, the participants were instructed to apply one of five strategies: (1) Physical Practice (PP), in which the participant is actually playing through the designated etude 3 times; (2) Mental Imagery (MP), in which the participant auditorily and kinesthetically imagines the music 3 times; (3) Singing (SOL), in which the participant sings the music, optionally using solfege 3 times; (4) a combination of SOL, PP, and MP (COM), in which each strategy was applied once in the given order; (5) a no practice (NP) control strategy, in which the participant read an unrelated article for an equivalent amount of time (approx. 3 min). The metronome was not used during practicing. Specifications on how to carry out the particular strategy were provided in written instructions (see Supplementary Material, Appendix 1).

In order to experimentally control and monitor the practice sessions and which strategies were used for optimal comparison, we deliberately instructed the participants to play through the etudes and/or sing/imagine according to the applied strategy a specific number of times, acknowledging the obvious fact that this is not how many musicians typically practice. This is in accordance with a similar study by Ross (1985), in which trombone students performed the etudes 3 times with each strategy.

The order of the etudes, as well as the order of the practice strategies, was randomized using randomize.org to exclude potential effects arising from the order (e.g., attention span and exhaustion), as well as unwanted effects due to remaining differences in difficulty between the etudes (An illustration of the experimental setup is provided in Figure 2).

The experimental sessions as well as the recordings were carried out by a research assistant who had no part in the data analysis (sound files and statistics). The experimenter was silently present in the room throughout the complete session to monitor the procedure and observe whether the participant was using the correct strategy, number of repetitions, preventing potential movement of body-parts during the imagery strategy and whether solfege was used during the singing strategy. Performances were recorded in high quality uncompressed digital audio using a Zoom H6^®^ recorder, placed 2 m away from the participant and 1.5 m above the floor, with the participant being seated. To create sound files for subsequent assessment and analysis, all recordings were edited in Adobe Audition CC 2018 and saved in wave-file format at 24-bit resolution and 44.1 kHz sample rate. The process yielded 498 samples, 249 T1-files representing pre-acquisition performances and 249 T2-files representing post-intervention performances. Two files were discarded due to one participant accidentally repeating one strategy.

### Performance Assessment

All recorded performances were independently assessed by three raters who were all professional orchestral trumpet players. Two raters held full time positions in a symphony orchestra and one worked as a freelance trumpet player. None of the raters were involved in teaching the participants. The raters were required to assess the recorded performances on five different parameters: (1) pitch errors, (2) rhythm errors, (3) sound quality, (4) intonation, and (5) musical expression. Pitch and rhythm errors were measured by counting the number of bars in which pitch and rhythm differed from the musical score. General sound quality in each performance, intonation and musical expression were rated on a Likert type scale with 1 representing the best and 5 representing the worst quality level. The term pitch was defined as tones within half-step (or chromatic) deviations, whereas the term intonation meant fine-tuning of less than one half-step. Sound quality, intonation and musical expression were assessed by the expert raters’ subjective judgment with no further instruction. Spearman rank correlation analyses showed a high inter-rater agreement for ratings of percentage of bars with pitch errors, percentage of bars with rhythm errors, sound quality, intonation, and musical expression (see Table 4).

**TABLE 2:** Effect sizes (*r* = Z/√n, based on *Z*-values from the Wilcoxon signed rank tests and the sample size, *n* = 50) for the change from T1 to T2 are shown for each practice strategy and rated parameter.

Strategy	Pitch errors	Rhythm errors	Sound quality	Intonation	Musical expression
PP	0.78***	0.75***	0.53***	0.49***	0.62***
COM	0.64***	0.70***	0.49***	0.48***	0.69***
SOL	0.60***	0.66***	0.48***	0.44**	0.51***
MP	0.67***	0.70***	0.25	0.41**	0.45**
NP	0.54***	0.63***	0.29*	0.21	0.42**

***p < 0.001, **p < 0.01, and *p < 0.05.

**TABLE 3:** Mean improvement in percentage after application of the five practice strategies as measured on five parameters.

Strategy	Pitch errors	Rhythm errors	Sound quality	Intonation	Musical expression	Average
PP	38.7% (38.2%)	44.1% (52.1%)	12.5% (25.6%)	10.5% (33.0%)	15.9% (22.7%)	24.3% (34.3%)
COM	34.2% (46.6%)	45.7% (56.0%)	12.9% (30.4%)	12.0% (31.3%)	21.9% (28.2%)	25.3% (38.5%)
SOL	27.7% (47.0%)	32.4% (48.2%)	10.5% (33.6%)	10.0% (31.5%)	11.9% (25.0%)	18.5% (37.1%)
MP	27.6% (44.6%)	38.3% (43.8%)	1.4% (36.8%)	5.3% (28.8%)	7.0% (40.9%)	15.9% (39.0%)
NP	5.8% (73.0%)	28.2% (48.2%)	3.4% (28.4%)	1.3% (26.5%)	7.0% (29.1%)	9.1% (41.0%)

*Standard deviation is shown in parenthesis.*

**TABLE 4:** Inter-rater agreement.

Ratings	Jury member agreement (*r_*s*_)*			Jury agreement with CUEX (*r_*s*_)*		
	#1 - #2	#1 - #3	#2 - #3	#1 - CUEX	#2 - CUEX	#3 - CUEX
Percentage of bars with pitch errors	0.87***	0.88***	0.87***	0.69***	0.71***	0.69***
Percentage of bars with rhythm errors	0.84***	0.83***	0.86***	0.74***	0.79***	0.79***
Sound quality	0.60***	0.66***	0.54***	–	–	–
Intonation	0.52***	0.60***	0.52***	–	–	–
Musical expression	0.66***	0.67***	0.60***	–	–	–

Pairwise comparisons of jury members and CUEX ratings with Spearman’s rank correlation. *** indicates agreement significance at p < 0.001.

Raters listened to the performances using high-end headphones at a fixed volume and without knowledge of who was playing, which strategy had been used, and whether they were recorded before or after practicing. The files were pseudo-randomized to ensure that the jury members did not assess the recordings in the same order. To standardize the analyses, the number of bars with errors was adjusted by the total number of bars in each etude. This was achieved by calculating the percentage of bars with errors in each piece by dividing the number of bars with errors by the total number of bars in the respective pieces and multiplying by 100.

### Semi-Automatic Error Detection

To verify that the expert ratings of numbers of bars with pitch and rhythm errors were a true reflection of the actual number of notes with errors, we also performed a semi-automatic pitch and rhythm error detection procedure that provides pitch and onset times of each played note, as implemented in the CUEX software (Friberg et al., 2007). CUEX has shown high reliability in recognizing tones in human performances (91.8% correct tone detections) (Friberg et al., 2007). However, manual inspection and correction of the automatic detections was necessary to reliably identify each pitch and rhythm error. Due to the significant time consumption required for the manual corrections of the CUEX tone detection outcome, we limited the automatic error detection to a sub-sample of 100 audio files, all playing the same etude (representing all 5 practice strategies and T1 and T2 performances). All manual corrections were performed by an expert musician, who was blind to the training conditions represented in the audio files.

Using MATLAB (©1994-2020 The MathWorks, Inc.), each semi-automatically processed audio file was compared to a correct MIDI version of the melody taken from the musical score. For each detected note, the script tested whether the pitch deviated more than a semitone from the correct target note. Each time the pitch deviated more than a semitone it was defined as a pitch error. Cross-checking whether the detected pitch error was in fact due to an error in tone order (e.g., an extra note was played or a note was missing) involved testing whether keeping the played note, removing the played note (an extra note), or removing the correct note (a missing note), resulted in lower pitch deviation during the next 2 s playback. In addition, the automatic matching procedure was visually inspected and manually corrected whenever necessary. Finally, pitch errors deviating a semitone or more and rhythm errors with note durations deviating a 1/16 note (125 ms) or more were defined as errors and counted for statistical analyses.

### Statistical Analyses

Statistical analyses were conducted with SPSS Statistics version 27 (IBM, Armonk, New York, United States). Kolmogorov-Smirnov tests results with *p* = 0.000-0.200 and Shapiro-Wilk test with *p* = 0.000-0.515 indicated that the expert ratings were not normally distributed for each expert rater, practice strategy, time point (T1, T2), and musical dimension. Generally, the scores for pitch and rhythm errors were skewed toward a relatively low percentage of errors. The sound quality and musical expression ratings were biased toward relatively low performance ratings (with inverted scales, higher scores = better performance). The distribution of intonation ratings was mixed. Based on these observations, the expert ratings were analyzed with non-parametric statistics.

#### Effects of Practice Strategies on Performance Improvements

In the first step, the potential effect of the intervention (the difference between T1 and T2) was calculated for each participant, each practice strategy, and each expert jury member. This was performed by dividing the post-practice ratings by the pre-practice ratings, subtracting 1 to obtain only the difference in ratio, multiplying by 100 to derive the percentage change, and multiplying by –1 to invert the direction, so that higher percentage scores (instead of lower) indicate the percentage performance improvement.

The resulting percentage improvement scores were used to calculate a median improvement score across jury members for each study participant. For the purpose of visualization of the results, visual interpolation across the five-point Likert scales was achieved based on the mean results for these metrics across all study participants.

Effects of practice strategy on percentage performance improvement from T1 to T2 were tested with Friedman’s 1-way ANOVA for repeated measures. Comparison of the performance improvement between T1 and T2, and follow-up pairwise comparisons of the percentage performance improvement compared to the percentage improvement with no practice, were conducted with Wilcoxon signed rank tests.

#### Correlation With Reported Practice Routines

To investigate how the specific types of solfege, focus of attention, and use of voice strategies used by the students affected their performance improvement, we applied the Kruskal-Wallis test for between-subject effects. For *post hoc* comparisons, and also for testing effects of gender and use of meditation, two-sample between-subject comparisons were conducted with the Mann-Whitney *U*-test. Since only one student was inexperienced with singing, the effect of previous singing experience was excluded from the tests. For testing these effects with each study participant and for each musical parameter, an overall performance score was applied, which was the median rating across practice strategy and pre/post practice scores. Moreover, an overall percentage performance improvement score was applied for each study participant and for each musical parameter, which was the median percentage improvement across practice strategies.

The possible relationships between years of music training, hours of daily practice, years of studying solfege, hours of sleep, hours of naps between practice sessions, and the performance scores as well as the percentage performance improvements were tested with linear regressions.

The resulting *P*-values were reported without correction for multiple comparisons. However, for tests of the main hypotheses regarding the effect of the four active practice strategies on performance improvement, *P*-values were reported also with the Benjamini-Hochberg FDR correction (Benjamini and Hochberg, 1995).

## Results

### Effects of Practice Strategies on Performance Improvements

**FIGURE 3:**
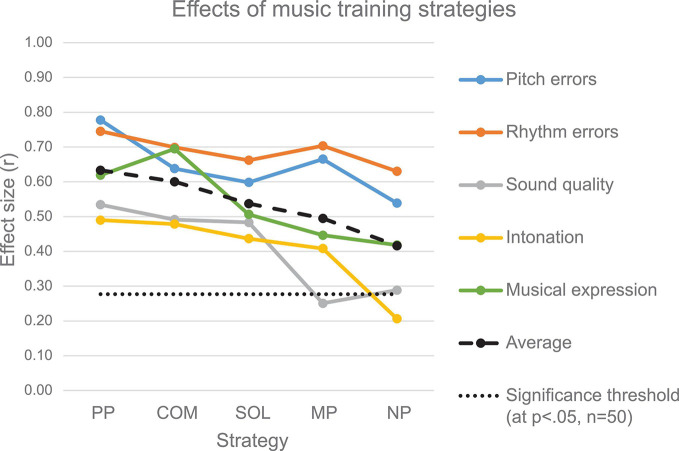
Improvement effects after application of the five practice strategies as measured on five parameters and on average across parameters. Effect sizes (*r* = Z/**√**n, based *Z*-values from the Wilcoxon signed rank tests) for the change from T1 to T2 are shown for each practice strategy and rated parameter. The effect sizes indicate the performance improvement in relation to the individual variance in the performance improvement. A higher effect size indicates a more consistent improvement across individual participants (The two-tailed significance threshold at *p* < 0.05, *n* = 50, is derived from the standard z-distribution as *r* = 1.96/**√**50 = 0.28).

As stated in Table 1 the participants were to various degrees familiar with singing, auditory and motor imagery as well as physical practice and were able to apply these strategies during the experiment. All practice strategies resulted in improvements with regard to pitch and rhythm errors (Figure 3 and Table 2). The sound quality improved with physical practice (PP), combined practice (COM), singing (SOL) and marginally with no practice, but not with mental imagery (MP) alone. Intonation improved with all practice strategies, except with no practice. The musical expression improved with all practice strategies.

In the following, we report performance improvement for the active practice strategies in comparison with no practice (NP). It should be noted that the performance improvement scores were weighted by the individual T1-performance score, thereby taking the individual performance level into account (e.g., a reduction of 8% to 4% bars with pitch errors is regarded as a 50% improvement, as is a reduction of 50% to 25% bars with pitch errors). By contrast, the results reported in Figure 3 and Table 2 show the T1 and T2 performance ratings without individual weighting. Therefore, the above and the following results are not directly comparable.

### Overall Improvement Across Musical Parameters

**FIGURE 4:**
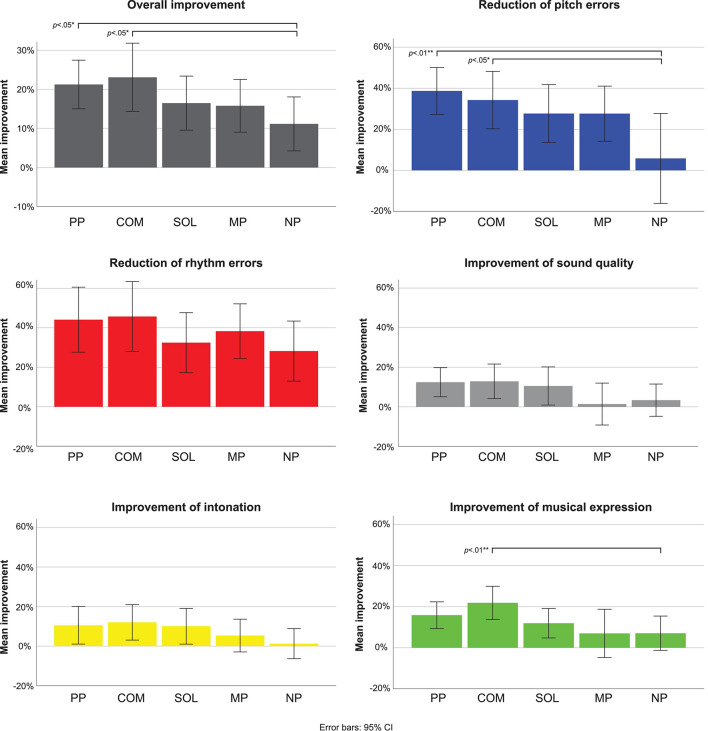
Mean overall improvement and improvement in pitch, rhythm, sound quality, intonation, and musical expression for the five different practice strategies. *p < 0.05; **p < 0.01.

The Friedman ANOVA showed that overall improvement differed depending on the practice strategy (χ2_F_(4) = 9.62, *n* = 49, *p* = *0.047*) (Figure 4). Wilcoxon signed rank tests showed that across the five tested musical factors, both PP (*z* = 2.33, *r* = 0.33, *p* = 0.020) and COM (*z* = 2.35, *r* = 0.33, *p* = 0.019) improved the overall performance in comparison to NP (Figure 4). No advantage of SOL (*z* = 1.14, *r* = 0.16, *p* = 0.256) or MP (*z* = 0.95, *r* = 0.14, *p* = 0.341) was found in comparison to NP.

#### Improvement According to Musical Parameters

Here, in accordance with our hypotheses, we report which specific musical parameters were significantly improved with each practice strategy relative to NP (Figure 4) (for a summary, see Table 3).

#### Pitch

Pitch accuracy improvement differed significantly between the applied practice strategies (χ2_F_(4) = 9.49, *n* = 45, *p* = 0.050) (Figure 4). Both PP (*z* = 2.84, *r* = 0.42, *p* = 0.004, *p*_FDR_^1^ = 0.022) and COM (*z* = 2.43, *r* = 0.36, *p* = 0.015, *p*_FDR_ = 0.055) improved the pitch accuracy (i.e., reduced the number of pitch errors) significantly more than NP. No significant differences in the improvement of pitch accuracy were found for SOL (*z* = 0.1.16, *r* = 0.17, *p* = 0.248, *p*_FDR_ = 0.341) and MP (*z* = 1.36, *r* = 0.20, *p* = 0.175, *p*_FDR_ = 0.275), when compared to the NP improvement.

#### Rhythm, Sound Quality, and Intonation

No significant differences between practice strategies were found on improvement of rhythm accuracy (χ2_F_(4) = 7.22, *n* = 41, *p* = 0.125, *p*_FDR_ = 0.275), sound quality(χ2_F_(4) = 6.87, *n* = 49, *p* = 0.143, *p*_FDR_ = 0.262), and intonation(χ2_F_(4) = 5.26, *n* = 49, *p* = 0.262, *p*_FDR_ = 0.320). There were, however, trends toward higher improvement gains in rhythm, sound quality, and intonation with PP and COM in comparison to NP (Figure 4 and Table 3).

#### Musical Expression

Improvement in musical expression depended significantly on the applied practice strategy (Figure 4), χ2F(4) = 12.46, *n* = 49, *p* = 0.014. Only COM showed a significantly higher improvement in comparison to NP (*z* = 3.05, *r* = 0.44, *p* = 0.002, *p*_FDR_ = 0.022). (PP: *z* = 1.63, *r* = 0.23, *p* = 0.103, *p*_FDR_ = 0.283; SOL: *z* = 0.54, *r* = 0.08, *p* = 0.588, *p*_FDR_ = 0.647; MP: *z* = 0.14, *r* = 0.02, *p* = 0.887, *p*_FDR_ = 0.887).

### Correlation With Reported Practice Routines

**FIGURE 5:**
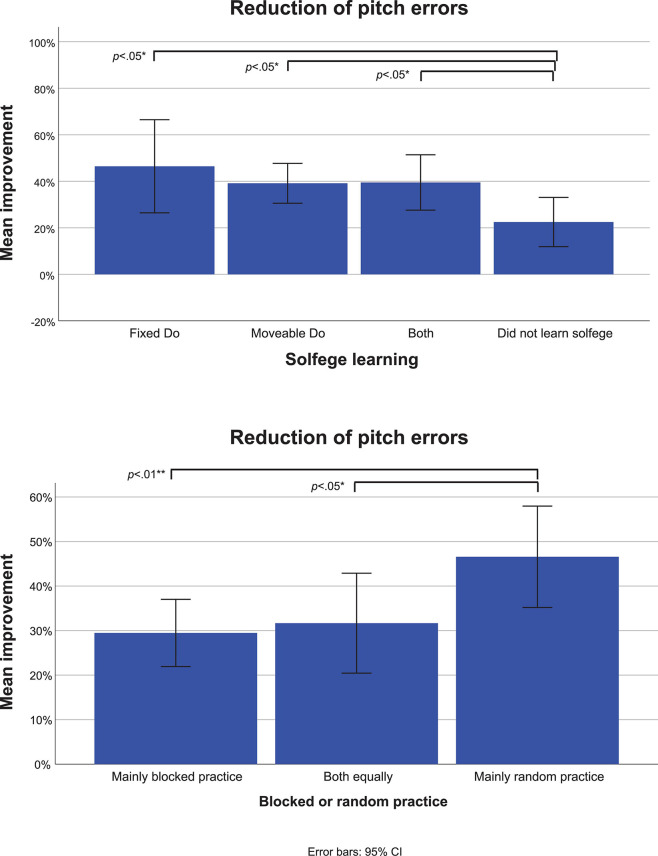
Effects of reported learning of different solfege and blocked vs. random practice methods on pitch accuracy improvement. *p < 0.05; **p < 0.01.

Kruskal-Wallis tests showed that the reduction of pitch errors differed depending on whether students had previously received solfege training (H(3) = 8.77, *n* = 50, *p* = 0.033) (Figure 5). Fewer pitch errors were observed for students who reported having learned the fixed Do (*n* = 5) (*z* = 2.30, *r* = 0.32, *p* = 0.023), the moveable Do (*n* = 17) (*z* = 2.21, *r* = 0.31, *p* = 0.025), and both methods (*n* = 12) (*z* = 2.20, *r* = 0.31, *p* = 0.029) in comparison to students who reported not having learned solfege (*n* = 16). In addition, reported use of *random* practice (*n* = 13) appeared to result in higher reduction of pitch errors as compared to reported use of *blocked* practice (*n* = 21) (*z* = 2.79, *r* = 0.39, *p* = 0.005) and use of a *combination of blocked and random* practice (*n* = 16) (*z* = 2.21, *r* = 0.31, *p* = 0.027) (Figure 5).

To verify that the low number of pitch errors was uniquely related to the use of solfege and random practice, multinomial logistic regression was applied to test whether these practice routines varied independently across the participants. The found effects of using solfege and random practice seemed independent, since the odds ratio of using random practice in relation to either blocked practice or a combination of random and blocked practice did not depend on the solfege strategy (R^2^ = 0.14 (Cox & Snell), 0.16 (Nagelkerke); model χ2 = 7.62, *p* = 0.267; neither the odds ratio of using random practice in relation to blocked practice, *p* = 0.315, nor the odds ratio of using random practice in relation to both random and blocked practice, *p* = 0.638, depended on the whether the participants did or did not learn solfege).

Furthermore, for students practicing meditation (*n* = 13) there were tendencies of lower performance improvements regarding rhythm errors (*z* = –2.15, *r* = –0.30, *p* = 0.031) and intonation (*z* = –2.7, *r* = –0.38, *p* = 0.007) in comparison to students not practicing meditation (*n* = 37).

Duration of music training (any music training, trumpet or cornet, other instruments), early onset (before the age of 7), hours of daily practice, accumulated hours of music playing (years of training × 365 × hours daily practice) in childhood and adolescence and years of solfege practice did not significantly relate to neither the performance improvement scores or the overall performance scores. Also, there were no significant effects of using singing voice to learn, internal/external focus of attention, constant vs. varied practice, possession of absolute pitch or experience with using imagery.

The parameters *hours of sleep* and *hours of naps between practice sessions* did not significantly relate to neither the performance improvements nor the overall performance scores for any of the five measured musical parameters.

Furthermore, we found no significant gender-related differences in neither the performance improvements nor the overall performance scores for any of the five measured musical parameters.

### Inter-Rater Agreement

**FIGURE 6:**
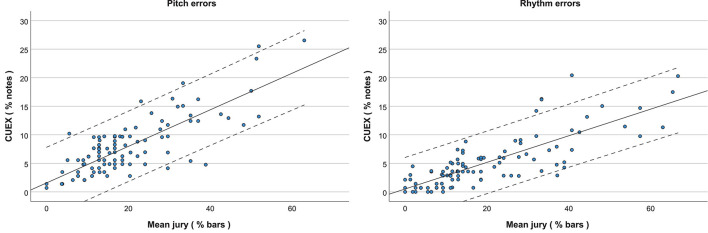
Regression plot showing the accordance between the expert jury’s assessment of bars with errors and the CUEX semi-automatic detection of single-note errors for Pitch (left) and Rhythm (right). The strong correlations show that the fast jury judgments of the percentage of bars with errors are appropriate substitutes for the slow fine-grained counting of the percentage of individual notes with errors. Dashed lines indicate 95% confidence intervals.

The Spearman rank correlation analysis showed that the inter-rater agreement between all jury members was high for ratings of percentage of bars with pitch errors, percentage of bars with rhythm errors, sound quality, intonation, and musical expression (see Table 4). Also, the percentage of bars with pitch and rhythm errors counted by the jury members reflected the percentage of notes with pitch and rhythm errors detected with the semi-automatic CUEX analysis (see Figure 6 and Table 4).

## Discussion

This study investigated the efficiency of five different practice strategies on the initial learning of an unfamiliar musical piece in an experiment involving 50 advanced trumpet students, as measured by ratings from three raters. Interestingly, all of the strategies, including the NP strategy, in which the participants did not practice but simply played the piece a second time, yielded progress in rhythm and pitch accuracy, reflecting the very high level of expertise of the participants. However, for the overall musical reproduction and quality, and in accordance with our hypothesis, both the practice strategy which combined equal amounts of physical practice, singing and imagery, and the strategy which involved physical practice for the same amount of time, generated improvements that were significantly higher than those of the no practice strategy. Likewise, and more specifically, the combined strategy was as efficient as physical practice in improving the pitch accuracy. None of the two effects were found for the practice strategies that relied on motor/auditory imagery or overt singing/solfege alone. The result is in accordance with prior studies (Ross, 1985; Coffman, 1990; Pascual-Leone et al., 1995; Bernardi et al., 2013), with the important difference that, as opposed to those studies, the combined strategy in the present study included overt singing.

Neither PP, COM, SOL or MP were significantly more effective than no practice in reducing rhythm errors and improving sound quality and intonation, although PP and COM showed trends toward higher gains. Remarkably, however and exceeding our expectations, the combined practice strategy produced a significantly higher level of musical expression as compared to all of the other four practice strategies. In addition, the results suggest that previous solfege training was a predictive factor for improvement in pitch accuracy. Similarly, incorporation of *random* practice strategies in daily practice routines yielded larger improvement gains compared to those using *blocked* practice. Contrary to common wisdom, duration and onset of music training, number of hours of daily practice and accumulated hours of music training did not significantly relate to neither the performance improvement scores nor the overall performance scores on any of the five measured musical parameters.

### Overall Musical Improvement

The implication that the complex task of learning to play a new piece of music on trumpet with high accuracy and quality can be achieved as efficiently by an equal mix of singing, imagining and playing as by repeated playing is remarkable, as the mixed strategy amounts to only one third of physical rehearsal with the instrument. As described above, current-day classical players and students often need to undertake highly intensified practice schemes, potentially risking damage to their physical apparatus and mental wellbeing. Thus, the finding that a diversified practice approach, which may be less exhausting and more satisfying, can yield a result that is similar to that of physical practice is highly encouraging for music pedagogy.

As the present study only involved advanced trumpet students, two questions remain: (1) whether these results are generalizable for beginner and intermediate students with less advanced procedural learning established, and (2) whether similar results could be expected for all practicing musicians. Pascual-Leone et al. (1995) found gains for beginner pianists in a strategy combining physical practice with mental practice compared with controls; Bernardi et al. (2013) found gains in advanced pianists using mental practice but to lesser degree than with physical practice; Coffman (1990) found significant gains in intermediate piano students using a combined strategy compared to mental practice alone and equal to physical practice and finally, Ross (1985) showed gains in trombone players using a combined strategy. Based on these previous findings, we have reason to believe that the results of the present study may be generalizable to learners of both different skill levels and different instruments and may reflect general principles of short term motor learning.

### Pitch Improvement and Singing

The rationale for seeing an improved pitch accuracy as a result of a singing based practice may partly be due to the nature of brass instruments. Brass players differ from other instrumentalists, including woodwind players, as they vibrate their lips to control the pitch of their instrument. Other instruments would strike or pluck a string, depress keys on a keyboard, or vibrate a wooden or double reed to initiate vibration, as is the case with woodwinds. Moreover, the brass players’ lip vibration must be very close to the resonance frequency of the air column, whose length is constantly altered through application of different valve combinations or slide positions within the instrument, which requires the player to carefully adjust lip muscle tension (Fletcher, 2000). This precision is similar to a singer’s control of the vocal fold vibration patterns, which has given rise to methods stressing the importance of mentally singing while playing as well as practicing the use of the singing voice, with or without solfege, in preparation of learning a piece of music. This is part of the foundation of the pedagogy of one of the most important teachers of brass instruments in the 20^th^ century, Arnold Jacobs, in formulating his concept Song and Wind (Frederiksen, 1996).

The potential impact of using the singing approach when practicing brass instruments might be supported by the observed improvement in pitch accuracy, resulting from the strategy in which singing was used in combination with physical practice and mental imagery. However, when practicing overt singing only, the participants in the study did not significantly improve their pitch accuracy more than when not practicing at all Several factors may explain this finding. Firstly, the participants’ experience with singing may have been insufficient or too diverse to drive an effect. Secondly, the duration of the intervention (approximately 3 min) might have been too short for an effect to materialize. This is in line with an observation by Haueisen and Knösche (2001) who reported absence of motor activation during listening to unknown pieces. Third, a potential effect of solfege may be suppressed by the fact that only 14 participants applied that method.

### Pitch Improvement and the Audio-Motor Loop

There may also be a neurobiological explanation of the benefit of adding auditory/motor imagery and overt singing to physical practice. The neural connectivity between auditory and motor areas, the audio-motor loop, is strongly established in trained musicians. For instance, primary motor regions corresponding to the finger that would have pressed the key for the given note become active when pianists listen to pieces they already know how to perform (Haueisen and Knösche, 2001), which is not the case with unfamiliar pieces. Conversely, watching a pianist playing in silence can evoke activation of auditory areas in piano players (Zatorre and Halpern, 2005). Consequently, auditory/motor imagery and overt singing may stimulate activity in auditory areas that allows for an off-line rehearsal of motor sequence control, thus leading to fewer pitch errors.

Bangert et al. (2006) compared professional pianists and non-musicians who either listened passively to short piano melodies or pressed keys on a mute MRI-compliant piano keyboard. When comparing activity in the observed cortical networks during the acoustic and the mute motion-related task respectively, the authors found a distinct increase exclusively in the pianists. Thus, despite quite different circuitries being involved, an integration of imagery of related musical sounds and movement is possible. A similar study showed how the extent of activity in auditory and motor areas was clearly increased by imagination of the absent modality (Baumann et al., 2007).

As mentioned in the Introduction, Gebel et al. (2013) studied trained pianists and trumpet players and found evidence for the existence of an auditory-motor loop in motor tasks. A study involving trained and beginner pianists (Bangert and Altenmüller, 2003) exhibited the effect of auditory-sensorimotor coactivation already emerging in the first few minutes of training and being firmly established within a few weeks. The evidence conveyed in these studies support the beneficial effect of combining imagery, singing and physical practice.

### Motor Imagery

The significant progress in pitch accuracy found with the use of the combined practice strategy may also be explained by the inclusion of motor imagery. Pascual-Leone et al. (1995) tested beginner pianists training a right-hand finger sequence for 2 h per day over 5 days. One group would practice physically, and another group would practice using motor imagery. In conclusion, the involvement of both physical practice and motor imagery seems to produce results that are equivalent to physical practice alone. However, because of the reduced application of muscle contractions, a combination of physical and mental practice may display a more economical use of the muscles involved, thereby being a less physically taxing alternative to physical practice. This finding is partly corroborated by the current study, where the combination of auditory/motor imagery, singing and physical practice is no less efficient than physical practice alone at improving pitch accuracy.

In the study by Ross (1985), involving 30 college trombonists of North American universities, the combination of physical and imagery practice strategies yielded significantly higher gains than any of the other strategies. While we in our study found a significant effect of the combined strategy on pitch accuracy, the strategy did not outperform physical practice, as was the case in the Ross study.

### Improvement in Musical Expression

In addition to a significant improvement of pitch accuracy, the combination of strategies improved musical expression to a significantly higher degree than no practice, an effect not found with other strategies, even physical practice. This is in accordance with a study by Kleber et al. (2007), involving professional singers who displayed increased activity in areas responsible for emotional processes during imagery. Using functional MRI-scanning, Kleber and colleagues found enhanced activity in areas responsible for emotions (ventrolateral prefrontal cortex and anterior cingulate cortex) in professional classical singers while they were imagining singing an Italian aria, as well as activated sensorimotor regions required for overtly performing the task. This suggests (i) an enhanced expressive involvement during imagery and (ii) demonstrates the involvement of the motor control network. Kleber et al. (2007) conclude that *“imagery “frees” us from the chains of external perceptual cues. Areas processing emotions also showed enhanced activation during imagined singing, which may reflect increased emotional recall during this task.”*

According to a review on fMRI-studies on mental imagery, no fMRI-study on professional instrumentalists has reported activation of emotional areas during processing of musical performance or kinesthetic imagery (Lotze, 2013). One explanation for the finding of increased musical expression in our behavioral study could be that brass players share similarities with vocalists who utilize vocal cords to initiate vibration. Analogically, brass players, as the only type of instrumentalists, must control pitch by vibrating part of their own anatomy, their lips, to excite the fundamental and corresponding partials in the air column of the instrument. Additionally, both groups of musicians share the use of respiratory muscles and the mouth area, including the tongue.

In our study, overt singing came out as the third most efficient strategy to enhance musical expression (Figure 4) when applied by itself, although not significantly better than the no practice condition. This was also true for average improvement across all parameters (dashed black line in Figure 3). Very few studies have explored this practice strategy and with inconclusive results, possibly owing to the participants’ different competency and familiarity with singing. One study by Davis (1981) found that structured singing activities are effective for supporting the development of instrumental performance skills, whereas another study by Rosenthal et al. (1988) observed that singing alone offered no immediate improvement over sight-reading in the assessed parameters, including pitch, rhythm, articulation, phrasing and dynamics. However, it was informally noted that the singing group of the study seemed uncomfortable with the task due to insufficient vocal competence, and that “singing may be more helpful for improving overall musicianship.” A smaller study (Ohsawa, 2009), involving 7 novice learners of piano, reported improvement in singing over non-singing conditions in students who were experienced in singing and the opposite, when unfamiliar with singing.

In the present study we found no effect of reported use of singing voice as an isolated practice strategy. However, we did find reduced pitch errors in students who reported to have learned solfege as opposed to students who did not learn solfege (Figure 5), with the fixed-Do approach showing a trend toward being more efficient than moveable-Do and learning both. This is corroborated by Apostolaki (2012), who found a positive effect of fixed-Do solfege. In his study, however, this was measured as improvement in aural skills, while our study illuminates how solfege may benefit main instrument performance.

### Solfege and Absolute Pitch

Surprisingly, during application of the SOL strategy, only 14 of the 32 participants who reported having learned solfege chose to use fixed-Do solfege. No significant improvement, however, was found in this specific group as compared to participants who sang without the use of solfege. Likewise, we observed no significant advantage of possession of absolute pitch, neither in terms of improvement nor on level of performance.

### Improvement in Learning Due to Elements of Random and Varied Practice

In the present study the COM condition offered a random practice approach which may partly explain the strategy’s added efficacy. In support of this argument is the fact that students who reported the frequent use of random practice exhibited an improvement in pitch accuracy, not found in students who mainly used blocked practice or both (Figure 5). This phenomenon could be partly explained by the increased involvement of dorsolateral prefrontal cortex in interleaved/random practice (Lin et al., 2013). This area is believed to be an essential component of the neural network responsible for procedural learning (Pascual-Leone et al., 1996) as it has extensive interconnections with regions involved in motor functions (Diamond, 2000; Lage et al., 2015), and it is also possible that this familiarity with random practice could prepare the participant for the rapid technique changes in the COM condition.

### Focus of Attention

A substantial number of studies (see especially Wulf (2013) review of 68 studies across different athletic and musical disciplines; Zachry et al., 2005; Zentgraf et al., 2009; Duke et al., 2011; Mornell and Wulf, 2019) have investigated the influence of focus of attention on training and performance outcomes and the vast majority, whether in sports or musical performance, points to external focus as being most efficient. The present study did not show any significant improvement relating to direction of focus, partly owing to the fact that only three participants reported mainly using internal focus and therefore yielded little statistical value. However, the self-reported use of focus of attention did not relate specifically to acquisition and performance as required in the study but to a general state while practicing.

### Music Training

The participants’ self-reported data revealed that duration of music training (any music training, trumpet or cornet, other instruments), hours of daily practice, accumulated hours of music training (years of training × 365 × hours daily practice) and years of solfege practice did not significantly relate to neither the performance improvement scores or the general performance scores. The finding is consistent with Anders Ericsson et al. (1993) findings that the amount of time spent practicing does not qualify as being the sole determinant factor sufficient to achieve excellence. A number of reasons for this may be considered: whether practice has been deliberate; effect from neuro-active hormones, such as adrenalin, endorphins, dopamine and stress hormones that support neuroplastic adaptations (Altenmüller and Furuya, 2017); social factors such as family and community, influence from teachers, motivational and attentional factors (Wulf and Lewthwaite, 2016), and genetic influences.

Early start is considered an advantage in learning to play a musical instrument (Steele et al., 2013), as those individuals who later become professional musicians usually start their musical training very early, sometimes before the age of six, when the adaptability of the central nervous system is at its highest (Altenmüller and Furuya, 2017). Our study, however, provided no evidence of improvement nor general level in those who reported starting before the age of 7, compared to later starters.

### Sleep and Meditation

Sleep has been implicated as an important factor in learning and consolidation of motor skills (Walker et al., 2002; Nishida and Walker, 2007). The influence of sleep has also been shown in consolidation of motor skills in music (Allen, 2007) and in auditory learning (Gaab et al., 2004). We did not include sleep as a factor in our study as students were tested right after practice. However, the participants’ self-reported general sleep duration showed no significant relationship with neither performance improvements nor overall performance scores, which may be explained by one study (Tucker et al., 2016) suggesting that musicians have the capacity to consolidate a motor skill across waking hours. Meditation has also been seen to have a positive effect on motor learning (Immink, 2016), but surprisingly we found negative effects on improvement of rhythm and intonation in those who reported regular use of meditation compared to those who reported no use of meditation.

### Gender

As expected, we did not find any significant difference in improvement or general level of performance related to gender.

### Limitations of the Study

The study only measured short term improvements in a brief practice interval (approximately 3 min). It could have been interesting to have employed longer practice intervals and also to assess the long-term effects of the acquisitions. This, however, would have been a logistic challenge, considering the magnitude of the number of participants and their geographical diversity.

We did not measure the difference between auditory and motor imagery, since this would have required inclusion of a sixth strategy, adding significantly to the logistic complexity. However, prior to the imagery trial, the participants were instructed to practice utilizing both strategies simultaneously, as is often the approach. Observation of the potentially differentiated effect of these two strategies nonetheless might have provided an interesting aspect to the findings.

Finally, it is important to bear in mind that the correlation analyses between practice routines and performance improvement are based on a high number of exploratory tests, which should of course be taken into consideration when interpreting the findings.

## Conclusion

This study investigated the efficiency of complementary practice strategies on the initial early learning and short time gains of an unfamiliar musical piece in an experiment involving 50 trumpet students. The results confirm and extend previous findings showing that, compared to no practice, a strategy combining physical practice, imagery and singing was just as efficient as extensive and repetitive physical practice in improving both the overall performance and the pitch accuracy, and more efficient than practice strategies that relied on motor/auditory imagery or overt singing/solfege alone.

Moreover, the combined practice strategy produced a significantly higher level of musical expression as compared to all other four practice strategies. The results indicate that application of mental imagery and singing may have a strong potential as complementary practice strategies, providing a less physically taxing alternative to physical practice and a more musical outcome.

Furthermore, among the trumpet students who reported to have learned solfege, there was an improvement in pitch accuracy relative to students who did not learn solfege. A similar result was apparent in students who reported to mainly apply random or interleaved practice compared to students reporting applying mainly blocked practice or both. Years of music training, early start, amount of hours daily practice, accumulated hours of music training, however, did not significantly affect the extent to which short-term training increased performance improvement scores nor the general performance scores before and after the training session. In conclusion, the findings suggest that applying practice strategies that complement conventional physical practice can reduce physical practice quantity while maintaining the same performance quality. Furthermore, the study adds valuable insight into brass instrument performance, which may generalize to musical practice and, in a wider perspective, to many other forms of learning, in which cognitive processes and motor skills are involved.

## Data Availability Statement

The raw data supporting the conclusions of this article will be made available by the authors, without undue reservation.

## Ethics Statement

The studies involving human participants were reviewed and approved by Internal Review Board (IRB) at The Danish Neuroscience Center at Aarhus University, Denmark. Written informed consent from the participants’ legal guardian/next of kin was not required to participate in this study in accordance with the national legislation and the institutional requirements.

## Author Contributions

KS was the main author of the manuscript. NH carried out the data analysis and statistics. BK, PV, and BP were the co-authors of the manuscript. CC carried out the experiments. All authors contributed to the article and approved the submitted version.

## Conflict of Interest

The authors declare that the research was conducted in the absence of any commercial or financial relationships that could be construed as a potential conflict of interest.

## Publisher’s Note

All claims expressed in this article are solely those of the authors and do not necessarily represent those of their affiliated organizations, or those of the publisher, the editors and the reviewers. Any product that may be evaluated in this article, or claim that may be made by its manufacturer, is not guaranteed or endorsed by the publisher.

## Footnotes

1 with the Benjamini-Hochberg FDR correction.
