# Supplementary material for: Imagine, Sing, Play- Combined Mental, Vocal and Physical Practice Improves Musical Performance
Source: Front Psychol. 2021 Oct 25;12:757052. doi: 10.3389/fpsyg.2021.757052 (PMC8573425; doi:10.3389/fpsyg.2021.757052)

Appendix 1

After completion of the initial sight-reading T1-recording, the participant would be presented with one of the following written instructions, depending on which strategy had been determined in the randomization process. Up to this point the participants were oblivious to the type of practice they were required to apply.

Text to be presented to participant prior to PP:

1. Practice the excerpt 3 times by playing through at tempo of your own choice without a metronome trying to improve as much as possible.
2. Play the excerpt once through as well and as expressively as possible while being recorded at the designated tempo accompanied by metronome. Do not stop or go back to repeat any notes.

Text to be presented to participant prior to MP:

1. Mentally play the excerpt 3 times through at your own tempo without a metronome. Do not make any physical movements. Do not stop or go back to repeat any notes. Try to mentally hear the music being played, like pitch, timbre, phrasing, dynamics, expression, articulations etc., but do not vocalize. Try to imagine all physical aspects of playing, like embouchure, tongue, breathing and fingers, but do not do any actual movements.
2. Play the excerpt once through as well and as expressively as possible while being recorded at the designated tempo accompanied by metronome. Do not stop or go back to repeat any notes.

Text to be presented to participant prior to SOL:

1. You are to sing through the excerpt 3 times at your own tempo preferably using solfege. If you do not know solfege you can just sing using any syllable you want (this is not being recorded). Do not stop or go back to repeat any notes.
2. Play the excerpt once through as well and as expressively as possible while being recorded at the designated tempo accompanied by metronome. Do not stop or go back to repeat any notes.

Text to be presented to participant prior to COM:

1. Sing through the excerpt once, preferably using solfege. If you do not know solfege you can just sing using any syllable you want (this is not being recorded). Do not stop or go back to repeat any notes.
2. Practice (play) the excerpt at your own tempo once through.
3. Mentally play the excerpt once through at your own tempo without a metronome. Do not make any physical movements. Do not stop or go back to repeat any notes. Try to mentally hear the music being played, like pitch, timbre, phrasing, dynamics, expression, articulations etc., but do not vocalize. Try to imagine all physical aspects of playing, like embouchure, tongue, breathing and fingers, but do not do any actual movements.
4. Play the excerpt once through as well and as expressively as possible while being recorded at the designated tempo accompanied by metronome. Do not stop or go back to repeat any notes.

Text to be presented to participants prior to NP:

1. Read the indicated article for about 3 minutes.

2. Play the excerpt once through as well and as expressively as possible while being recorded at the designated tempo accompanied by metronome. Do not stop or go back to repeat any notes.

Appendix 2

Questions answered by participants regarding musical background, practice habits and strategies, life-style habits etc.

1. Which is your dominating hand?
2. At what age did you start any musical training?
3. At what age did you start playing the trumpet (or cornet)?
4. At what age did you start playing other instruments than trumpet (cornet)?
5. What other instruments have you played?
6. How many years have you done solfege or ear training?
7. Did you learn solfege using solmization (do-re-mi-fa-sol etc.)?
8. If you learned solfege, did you learn Fixed-Do (Do is always C) or Moveable Do (Do is always the tonic of the key)?
9. Do you possess perfect or absolute pitch (the ability to always accurately identify a pitch played on any instrument)?
10. Do you use your singing voice to learn trumpet repertoire as part of practicing?
11. How many hours do you generally practice daily (including mental practice, solfege, singing)?
12. How many hours did you play music daily (including band practice etc.) before the age of 8?
13. How many hours did you play music daily (including band practice etc.) between age 8 and 12?
14. How many hours did you play music daily (including band practice etc.) between age 12 and 17?

15. What percentage of your practice time is purely physical practice (actually playing the trumpet)?
16. What percentage of your practice time is purely mental practice? (Mental imagery, solfege, singing)
17. Do you do auditory imagery (imagining the sound of the music being rehearsed), motor imagery (imagining the movement of the muscles involved in playing, for instance lips, breathing muscles, fingers and tongue) or both?
18. How many years have you consciously practiced mental imagery?
19. How often do you do breathing exercises?
20. What percentage of your daily practice time is playing drills and exercises?
21. What percentage of your daily practice time is playing etudes?
22. What percentage of your daily practice time is playing solos (concertos etc.)?
23. What percentage of your daily practice time is playing orchestral excerpts?
24. Do you mainly do BLOCKED PRACTICE (longer sessions practicing one skill (1-1-1-1, 2-2-2-2, 3-3-3-3 etc.) or RANDOM PRACTICE (shifting between skills in shorter sections (1-2-3-4, 1-2-3-4, 1-2-3-4, etc.)
25. Do you mainly do CONSTANT PRACTICE (always practicing skills the same way) or VARIED PRACTICE (practicing each skill in several ways)?
26. Do you practice with INTERNAL FOCUS (focusing on different parts of the body and its muscles, i.e. lips, tongue, fingers, breathing muscles) or EXTERNAL FOCUS (focusing on the effect of playing, i.e. the music, sound of your instrument in the room, the wind going into the room)?
27. How much do you sleep daily including naps?
28. How often do you take naps between practice sessions?

29. Do you do any form of regular meditation?

30. If yes, what kind of meditation?

31. Do you do any form of regular exercise?

32. If yes, what kind of exercise?

Appendix 3

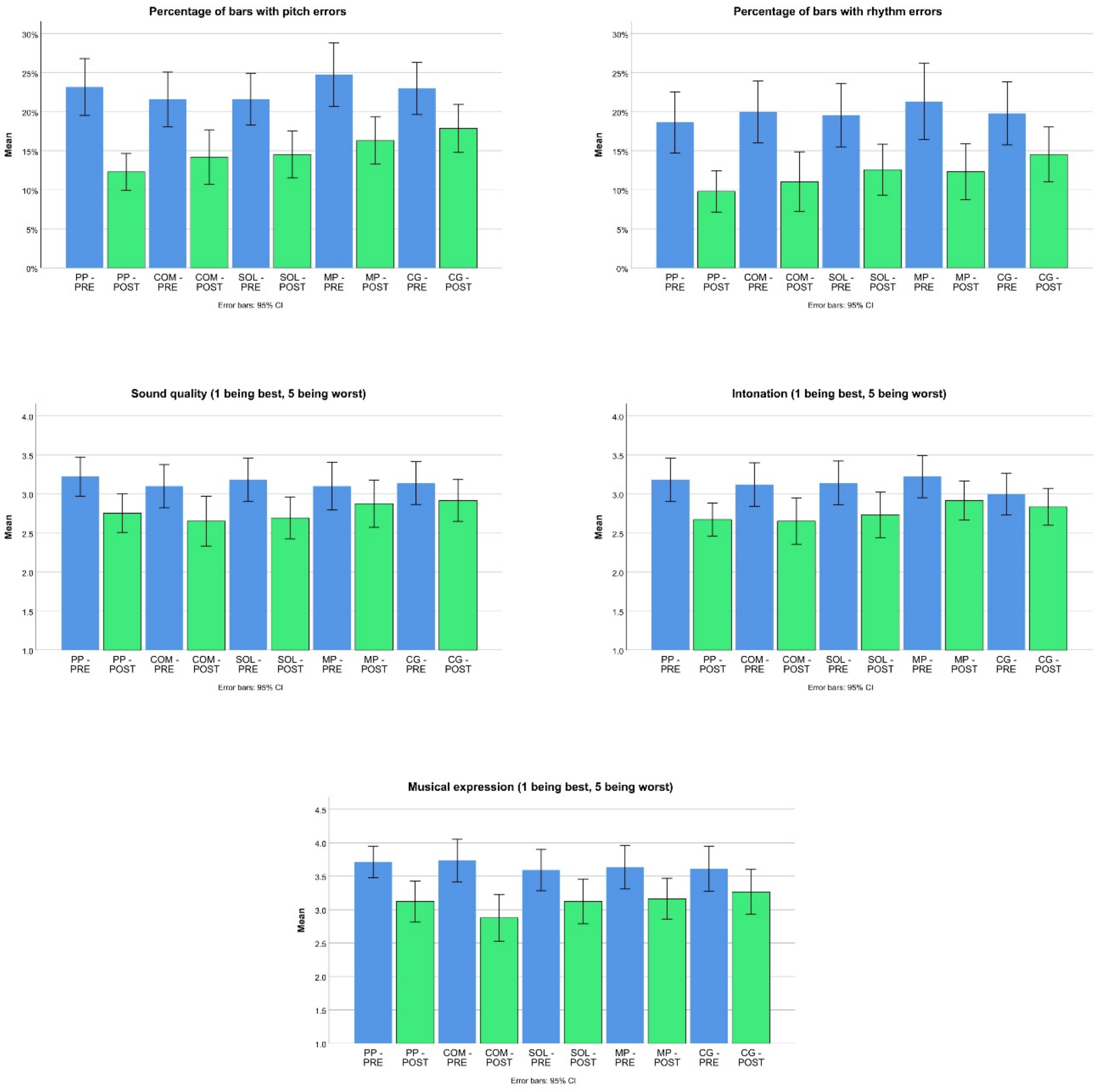

Mean performance ratings at T1 (blue) and T2 (green) 1692 shown for each of the five measured parameters and for each of the applied 1693 strategies. Error bars show 95% confidence intervals. PP=physical practice, 1694 COM=combined practice, SOL=solfege, and MP=mental practice.

Appendix 4

Trumpet in B♭

Practicing for Trumpet Players
Study #1

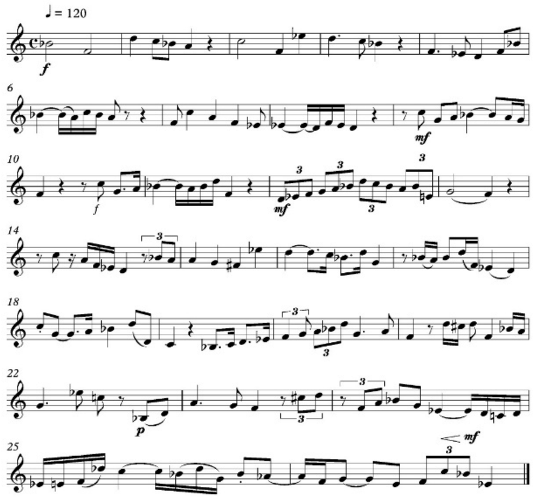

Trumpet in B♭

Practicing for Trumpet Players
Study #2

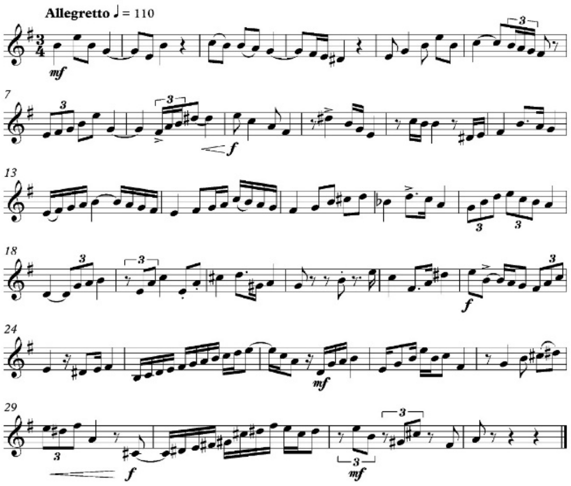

Trumpet in B♭

Practicing for Trumpet Players
Study #3

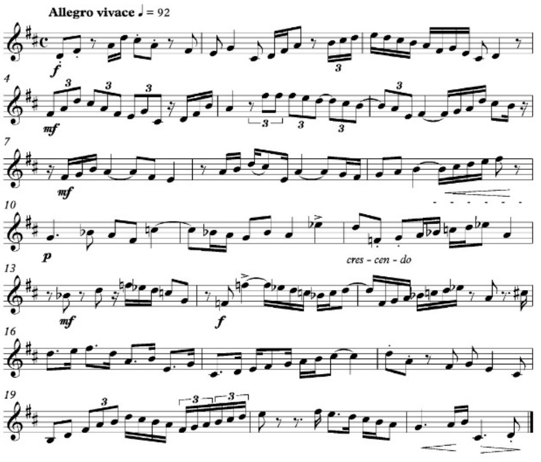

Trumpet in B♭

Practicing for Trumpet Players
Study #4

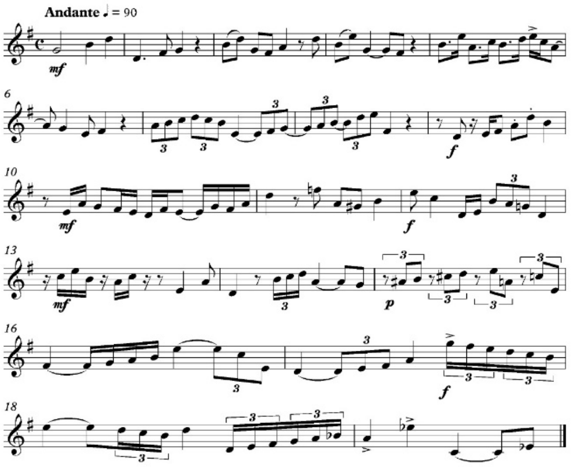

Trumpet in B♭

Practicing for Trumpet Players
Study #5

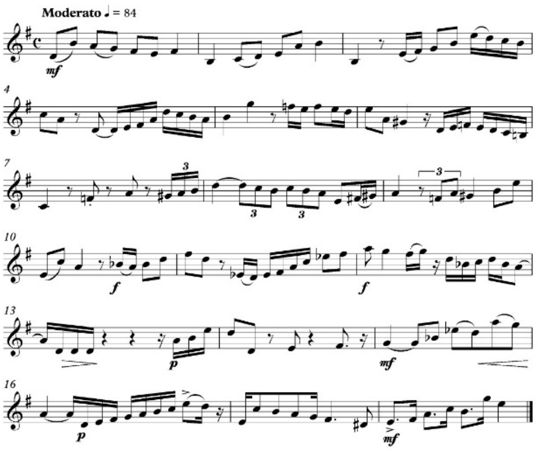

Supplement: Supplementary file 1 [file Data_Sheet_1.pdf]
